# Protocol: a medium-throughput method for determination of cellulose content from single stem pieces of *Arabidopsis thaliana*

**DOI:** 10.1186/s13007-015-0090-6

**Published:** 2015-10-12

**Authors:** Manoj Kumar, Simon Turner

**Affiliations:** Faculty of Life Sciences, University of Manchester, The Michael Smith Building, Oxford Road, Manchester, M13 9PT UK

**Keywords:** Cellulose assay, CESA proteins, irx mutants, High-throughput

## Abstract

**Background:**

Lignocellulosic biomass is an important renewable resource for biofuels and materials. How plants synthesise cellulose is not completely understood. It is known that cellulose synthase complex (CSCs) moving in the plasma membrane synthesise the cellulose. CESA proteins are the core components of CSC. In Arabidopsis, in vitro mutagenesis of proteins followed by complementation analysis of mutants lacking the gene represents an important tool for studying any biological process, including cellulose biosynthesis. Analysis of a large number of plants is crucial for these types of studies.

**Results:**

By using aspiration rather than centrifugation to remove liquids during various stages of protocol, we were able to increase the throughput of the method as well as minimise the sample loss. As a test case, we determined cellulose content of wild type and secondary wall *cesa* mutants across the length of primary shoot which was fond to be rather uniform in 7-week-old plants. Additionally, we found that the cellulose content of single mutants was comparable to the higher order mutants.

**Conclusions:**

Here we describe a medium-throughput adaptation of Updegraff’s method that allowed us to determine cellulose content of 200 samples each week.

## Background

Cellulose is the most abundant component of plant cell walls that constitute the majority of lignocellulosic material, an important feedstock for new generation of biofuels. Despite its importance, an understanding of how plants make cellulose is far from complete. Arabidopsis provides an excellent model system to study cellulose biosynthesis and a vast amount of progress has been made in the identification of genetic components of Arabidopsis cellulose biosynthesis machinery. Cellulose is synthesised by the cellulose synthase complex (CSC) particles moving in the plasma membrane [1–4]. The CESA proteins form the bulk of the CSC. Further studies on CSC will involve mutation of crucial residues of CESA proteins and studying their effect on the amount of cellulose in the cell walls. This will require analysis of a large number of plants.

Currently, most of the methods described for crystalline cellulose content determination are a variation of Updegraff method [5]. The method involves removing hemicellulose and lignin with acetic/nitric reagent [6]. Crystalline cellulose is resistant to acetic/nitric reagent but becomes disordered upon treatment with 67 % sulphuric acid making monomeric sugars available to be measured by a colorimetric method using anthrone as a dye [7]. The Updegraff method has previously been used for determination of cellulose content in Arabidopsis stem [1]. This involves preparation of alcohol insoluble residue (AIR) before acetic/nitric and 67 % sulphuric acid steps. However, most published studies have involved analysis of a few samples. Here, we report a practical adaptation enabling us to process up to 200 samples per week. As an example, we use the method the study cellulose content of single, double and triple mutants of secondary wall CESA genes.

## Cellulose content of *cesa* mutants

**Fig. 1:**
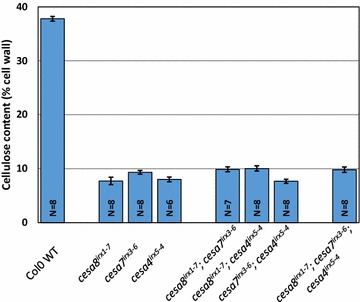
Cellulose content of SCW CESA single, double and triple mutants. The *error bars* are standard error of mean (SEM)

**Fig. 2:**
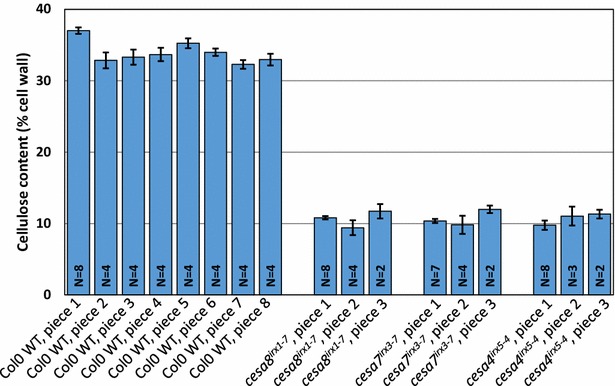
Cellulose content along the length of the inflorescence stem. For Col0, 40 cm long stem pieces were divided into eight pieces of 5 cm each while for the mutants, 15 cm long stems were divided into three pieces of 5 cm each. Piece 1 is closest to the rosette. All plants were 7-week-old. The *error bars* are standard error of mean (SEM)

**Fig. 3:**
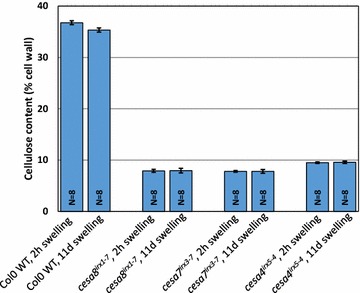
Effect of swelling time on cellulose content. The *error bars* are standard error of mean (SEM)

Our streamlined cellulose assay protocol allows a single person to process up to 200 samples in a week’s time with the help of only basic laboratory equipment. As an example, we analysed 200 samples from three separate experiments that involved comparing the cellulose content of single, double and triple *cesa* mutants (61 samples, Fig. 1); stem segments from multiple locations within the stem (76 samples, Fig. 2) and the effect of sulphuric acid treatment time (64 samples, Fig. 3).

Arabidopsis mutants that result from mutations in *CESA4*, *CESA7orCESA8* all exhibit multiple phenotypes including reduced cellulose content, reduced plant height and collapsed xylem [1, 8–10] and serve as excellent tools for studying cellulose biosynthesis in Arabidopsis. The three secondary cell wall CESAs form a complex and higher order mutants of secondary cell wall CESAs would be a valuable tool for advanced studies on the composition and structure of the complex. These higher order mutant combinations have not been described before. We crossed together the three single mutants to create three double mutants *cesa8*^*irx1*–*7*^*cesa7*^*irx3*–*6*^, *cesa8*^*irx1*–*7*^*cesa4*^*irx5*–*4*^ and *cesa7*^*irx3*–*6*^*cesa4*^*irx5*–*4*^ and the triple mutant *cesa8*^*irx1*–*7*^*cesa7*^*irx3*–*6*^*cesa4*^*irx5*–*4*^. There were no significant differences in the cellulose content of the single, double and triple mutants, all being around 10 % of cell wall material (Fig. 1). This is consistent with each of the three secondary cell wall CESA proteins being absolutely essential for cellulose synthesis in the secondary cell wall and with the fact that *cesa7*^*irx3*–*1*^ contains no cellulose in the secondary cell wall [11]. Any residual cellulose is likely to come from the primary cell wall and confirms that the CESA4, 7 and 8 have no role in primary cell wall biosynthesis.

Cellulose content of Ler0 WT plants has been shown to be increasing from 30 % for 26-day-old plants to 35 % for 36-day-old plants [1]. All the mutants used in this study are based on Col0 background which matures slower than Ler0. We chose to harvest the stem material for cellulose assays from 7- to 8-week-old plants. By this time, we expect the process of secondary cell wall deposition to be complete. To investigate whether this was the case we exploited the fact that the stem is a developmental series with the secondary cell wall deposition starting at the top. We divided the Col0 plants that were 40 cm tall into eight pieces of 5 cm each. Similarly, the *irx* mutants which grow to about 15 cm were divided into three pieces of 5 cm each. We found that at 7 weeks, all plants had mostly uniform cellulose content across the stem (Fig. 2). This is in contrast to when much younger plant stems are analysed that can exhibit a gradient of secondary cell wall deposition [12].

## Sources of variation in the cellulose assay protocol

### Sample loss

Previous applications of Updegraff method in Arabidopsis have involved fragmentation/homogenisation of stem material and subsequent centrifugation steps to collect the material after each treatment [1, 13]. This is a laborious process when large number of samples are involved. Also, during centrifugation, sometimes part of the stem material floats instead of settling into a tight pellet. This would result in loss of sample and inaccuracies in the final data. We kept the material as two pieces for each sample and removed all centrifugation steps. Instead, we used an aspirator to remove liquids after each step. The samples can be visually tracked as two pieces throughout the process until sulphuric acid treatment. Sample loss during aspiration can be minimised by using a drawn out Pasteur pipette to generate a narrower opening, however, should any sample be lost via aspiration, such samples are discarded. We typically started with eight replicates for each genotype and loss of one replicate would not affect the data. Step 11 of the protocol (see detailed protocol below) is the step most likely to cause the fragmentation of stem pieces because of larger liquid volumes involved.

### Factors affecting the anthrone assay

The basic aspects of the anthrone assay including the effects of anthrone heating temperature and heating time are discussed elsewhere [7]. However, as long as a set of standards is run with each anthrone batch and the samples within an anthrone batch are treated identically, most of the factors become insignificant.

### Effect of SA incubation time

**Fig. 4:**
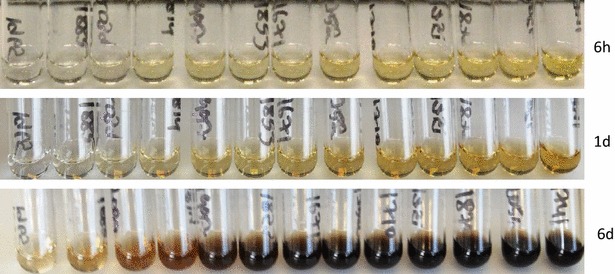
Effect of swelling time on sample colour. 13 tubes containing increasing amounts of cell wall derived glucose are shown. Acetic nitric extracted stem pieces were swelled in 67 % sulphuric acid and *left* on the bench. Photographs were taken 6 h, 1 day and 6 days after adding sulphuric acid. Normally, we carry out the anthrone assays within 8 h. The *error bars* are standard error of mean (SEM)

When a 50 mm Arabidopsis stem piece is treated with 67 % sulphuric acid, the resulting solution is largely clear for at least few hours after which it will start to go darker and will turn black eventually, depending upon the incubation time (Fig. 4). We compared cellulose content in a sample treated for 6 h with the one treated for 11 days and got identical results (Fig. 3). So extended incubation times with sulphuric acid is not an issue.

### Sulphuric acid age

We noticed that the stability of anthrone was dependent on the age of sulphuric acid. If we use acid from a 2 year old bottle, the colour of 0.3 % anthrone goes darker a lot quicker (within 10 min) as compared to acid from a fresh bottle which keeps the 0.3 % colour light yellow a lot longer (several hours).

## Further adaptations of the method

Recently, there have been methods published that report comprehensive analysis of cell wall components; for example Pettolino et al. [14] and Foster et al. [15]. Most of these methods usually involve relatively larger amounts of cell wall material which is ground into a fine powder. For example, Foster et al. [15] typically start with 60–70 mg dried AIR. Our analysis on the other hand typically stats with 10 mg stem material for wild type samples and 2 mg for *irx* mutants. The smaller amount of starting sample amounts and the deliberate avoiding of grinding steps to increase throughput means that not all of the side-analyses can be incorporated into our method. However, we do anticipate that an extraction of intact stems with 2 M trifluoroacetic acid (TFA) prior to acetic/nitric treatment can be performed to yield material for analysis of non-cellulosic polysaccharides with GC–MS [15].

The time of some steps could be further reduced with the use of additional equipment. For example, although our method means it is convenient to carryout the drying steps overnight; a vacuum desiccator or speed vac could be used for the drying steps to reduce time. Another potential time saving improvement could be performing the anthrone assay in a 96-well plate format. This will need an appropriate plate reader to be available which could be used with strong acids (67 % sulphuric acid). Additionally, it will also need 96-well plates that are resistant to 67 % acid at high temperature. However, both these variations have been previously used [15].

## Materials

### Reagents

- Pyrex glass tubes—16 × 100 mm, Scientific Laboratory Supplies, UK. 99449-16
- Corning^®^ phenolic cap, PTFE liner, Scientific Laboratory Supplies, UK. 9998-15
- 2 mL screw capped tubes—Starlab, UK. E1420-2330
- Caps for 2 mL screw capped tubes—Starlab, UK. E1480-100
- Acetic acid—Fisher Scientific UK Ltd. A/0400/PB17
- Acetone—Fisher Scientific UK Ltd. A/0600/PC17
- Ethanol—Fisher Scientific UK Ltd. E/0650DF/PB17
- Nitric acid—Fisher Scientific UK Ltd. N/2300/PB17
- Sulphuric acid—Fisher Scientific UK Ltd. S/9160/PB17
- Anthrone—Sigma. A1631-25G
- Glucose—Fisher Scientific UK Ltd. G/0500/61.

## Reagent setup

Acetic/nitric reagent—acetic acid: nitric acid: water, 8:1:2. To make 770 mL (enough to process 200 samples), add in order—560 mL glacial acetic acid, 140 mL water and 70 mL nitric acid.

- CAUTION. Strong smelling acid. Work in fume hood.

67 % Sulphuric acid—make 500 mL by slowly adding 335 mL conc. Sulphuric acid to 165 mL water.

- CAUTION. Concentrated strong acid. Wear appropriate gloves and work in fume hood. The bottle will get very hot; keep on ice and cool for at least 2 h. Work in fume.

Glucose standards—make 100 mg/mL glucose stock. Dilute to 10 mg/mL which can be used for preparation of a set of standards, 0–100 µg/mL. Use the following table.

**Table:** 

Standard name	Glucose conc (µg/mL)	Water (µL)	10 mg/mL glucose (µL)
S1	0	10,000	0
S2	10	9990	10
S3	20	9980	20
S4	40	9960	40
S5	60	9940	60
S6	80	9920	80
S7	100	9900	100

### Arabidopsis plants

Secondary cell wall CESA t-DNA mutants, *cesa4*^*irx5*–*4*^ (SALK_084627), *cesa7*^*irx3*–*6*^ (SAIL_24_B10), *cesa7*^*irx3*–*7*^ (GABI_819B03) and *cesa8*^*irx1*–*7*^ (GABI_339E12) were obtained from NASC and homozygous plants identified. Genetic crosses were made among the three single mutants to obtain all three possible double mutant combinations and the triple mutant. Homozygous plants were isolated for all the double mutants and the triple mutant.

Arabidopsis plants were first grown on ½ MS plates for 7 days in an incubator and then transplanted on a 1:1:5 mixture of perlite, vermiculite and compost. Plants were grown for a further 7 weeks on soil under long day conditions (16 h/8 h day/night, 22C/18C temperature and 80 % humidity). When plants were 7–8 weeks old, primary inflorescence stem was harvested by severing above the rosette level. Cellulose content was analysed with the detailed step by step protocol described below.

## Equipment

- Filter paper—Whatman. 1001090
- Foam floats—Heathrow Scientific. HS2165C
- Boiling water bath
- Aspirator
- Spectrophotometer
- Fume hood
- Stepper pipette
- Metal racks.

## Equipment setup

Aspirator trap setup—connect the trap outlet to a pressure device. To the inlet, attach a drawn out glass Pasteur pipette to ensure no sample could be “sucked in”.

## Procedure

### Material collection: TIMING up to 6 h for 200 plants

1. When plants are 7- to 8-week-old, harvest primary shoot of Arabidopsis by severing the plants just above the rosette level. Discard basal 5 mm and collect next 50 mm piece. Divide it into two roughly equal pieces and put in a 2 mL Eppendorf tube containing 1.5 mL of 70 % ethanol.
  PAUSE POINT. Samples can be stored in 70 % ethanol at room temperature until needed.

### Preparation of alcohol insoluble residue (AIR): TIMING up to 6 h for 200 samples

2. Incubate samples (in 70 % ethanol) at 70 °C for at least 1 h (mix by inversion after 30 min).
3. Remove 70 % ethanol using aspirator.
4. Add 1.5 mL 70 % ethanol and incubate at 70 °C for at least 45 min (mix by inversion after 30 min).
5. Add 1 mL of acetone and let the tubes stand for at least 2 min.
6. Aspirate off acetone and allow tubes to air-dry in a fume hood for 3–4 h. Tubes can be further dried by incubating in the 37 °C oven for overnight. Keep the tubes in racks covered with aluminium foil.
  PAUSE POINT. Dried AIR samples can be stored at room temperature until a later date when you are ready to perform next step.

### Weighting of AIR and transfer to glass tubes: TIMING up to 6 h for 200 samples

7. Determine dry weight of wall material (AIR). Dried stem pieces are strong enough to be handled with a pair of forceps; weigh them in weigh boats on a fine balance. After weighing transfer stem pieces to the pre-labelled glass tubes. Use metal racks to store the tubes (as in step 10, tubes will need incubation in a water bath). To save time do not cap the tubes. If they need storing, keep the tubes on racks and cover with cling film to avoid dust going in.
  PAUSE POINT. Weighted samples can be stored at room temperature until a later date when you are ready to perform next step.
8. At this step also include a filter paper sample (about 10 mg weight), which acts as a positive control.

### Acetic/nitric extraction: TIMING up to 8 h for 200 samples

**Fig. 5:**
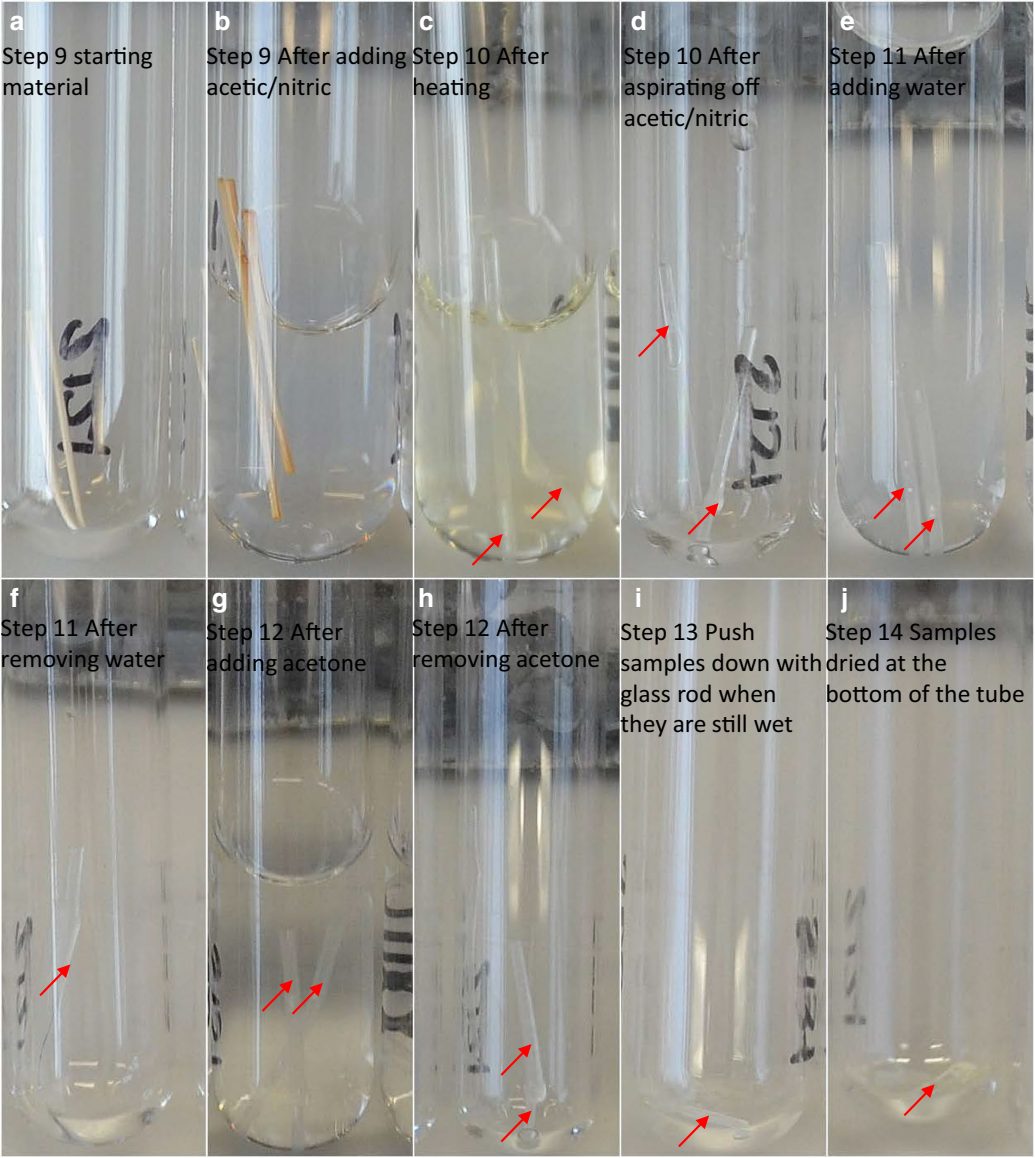
Sample processing through various stages of acetic/nitric treatment (**a**–**j**). One representative sample with a starting sample weight of 7 mg is shown (**a**). The wild type samples tend to be thicker and are much easier to track along the various steps. However, the *irx* mutant samples are comparatively thinner and they also tend to be more “wonky” making them relatively difficult to follow around during the aspiration steps. Also they go even more translucent than the wild samples making them more difficult to spot. So more care is needed in handling such samples. In step 13 (**i**), it is critical to push the samples down to the *bottom* of tube when they are still wet. Once dried at the *bottom* (**j**), they will be accessible to H_2_SO_4_ in step 16 (Fig. 3)

9. Carefully add 3 mL of acetic/nitric reagent to the wall material. Cap the tubes with PTFE seal caps. Photographs of four samples with a range of starting sample weight are shown in Fig. 5. These photographs show how the material becomes translucent and fragile after the acetic/nitric treatment.

  CAUTION. Work in a fume hood and wear appropriate PPE.
  CRITICAL STEP. Only use PTFE seal caps as the latex seals will disintegrate in the next step.
10. Place the tubes in a boiling water bath for 30 min. Allow to cool on bench. Aspirate off the acetic-nitric reagent. After this step, the samples become gelatinous and fragile, so handle carefully.
  CAUTION. Boiling water, work in a fume hood and use a long pair of forceps to take samples in and out of boiling water bath.
  Maintain water level in the bath.
  Discard acetic/nitric reagent appropriately.
11. Add 8 mL of water. Keep on bench for 15 min and then aspirate off the water.
  CRITICAL STEP. You are most likely to lose material during this step because larger volume makes the pieces float around more freely. Start aspirating slowly and let the pieces settle on the walls before removing all the liquid.
12. Add 4 mL of acetone. Let the tubes stand for 5 min and aspirate off the acetone.
13. After aspirating acetone, push the pieces down to the bottom of the tube using a blunt end glass rod.
  CRITICAL STEP. It is extremely important to push the pieces down when they are still wet. If they dry too high up in the glass tube, they will be inaccessible to the sulphuric acid in the next step.
14. Air-dry tubes in a fume hood for 3–4 h. They can be further dried by incubating in the 37 °C oven for overnight. Keep the tubes in racks covered with aluminium foil. After drying, samples can be stored until needed.

### Sulphuric acid swelling and dextran measurement: TIMING up to 8 h for 200 samples

15. At this step, we recommend arranging samples in order to ensure replicates of same genotype are spread across different batches in order to minimise any systematic errors.
16. Add 1 mL of 67 % sulphuric acid to each glass tube. Make sure all the material is covered by acid. Shake for 1 h at room temperature or until there are no visible material left. Vortex samples occasionally if needed. Only swell the number of samples you can handle during the day. Usually, samples are colourless for about 24 h before they start to go first brown and then black.
  CAUTION. Strong acid, wear appropriate PPE and work in the fume hood.
17. For paper controls, make the final volume up to 5 mL by adding 4 mL of 67 % sulphuric acid. This is to ensure that the OD values will fall within the standard curve. AIR samples remain 1 mL.
18. Aliquot out 500 µL of each glucose standard solution in a heat resistant, 2 mL screw-capped tube. For each test sample, label a tube and aliquot 500 µL of water into it.
19. Add 20 µL (out of a total of 1 mL) of swelled sample to 500 µL water. For paper control, use 20 µL (out of a total of 5 mL).
  CAUTION. Use a dedicated P20 pipette with filter tips as the acid can gradually damage the pipette making it inaccurate.
20. Prepare 0.3 % anthrone in conc. sulphuric acid. Mix well and keep on ice for at least 5 min. The anthrone solution should be light yellow in colour. It will go darker and darker with time but should be stable at least for few hours. Prepare anthrone solution in batches of about 100 mL. Always run a new set of standards with each anthrone batch.
  TROUBLESHOOTING.
21. Carefully add 1 mL of anthrone reagent to the side of the tube so that it sinks to the bottom without significant mixing with the sample. Once, anthrone has been added to all tubes, tightly cap tubes and mix thoroughly by inversion.
  CAUTION. After mixing anthrone reagent with water, tubes will get very hot, handle carefully. Wear suitable PPE for strong acid, handle in the fume hood.
22. Place the tubes in a boiling water bath for 5 min. Cool on ice.
  CRITICAL POINT. It is important to keep the temperature and duration of heating consistent for each batch of samples.
23. Transfer samples to disposable cuvettes and measure OD620. Make sure that the OD values for the test samples fall within the standard curve range.
  CAUTION. Discard liquids, plastic ware and glass tubes appropriately. PTFE sealed caps can be reused after rinsing with plenty of water and drying. The cuvettes are not completely acid resistant and should be discarded straight after use.
  TROUBLESHOOTING.
24. Calculate the glucose amount in each sample from OD620 values by using the standard curve regression line. Calculate cellulose content (% cell wall) with the following formula:
  Cellulose content (% cell wall) = amount of glucose in sample (µg)/cell wall weight from step 7 × 100 × total volume of sulphuric acid (µL) in step 18/20 (µL sample used in the anthrone assay).

## Anticipated results

The protocol described is very robust and produces reproducible results (Figs. 1, 2, 3). A 50 mm long Arabidopsis stem piece collected from 7- to 8-week-old plants will weigh 2–10 mg in step 7 depending upon the genotype of the plants. Pieces from wild type plants will weigh closer to 10 mg while the cellulose deficient mutants are around 2 mg. Considering that the wild type samples contain about 30 % cellulose and the cellulose deficient mutants about 10 % cellulose in their walls, using 20 µL sample in step 19 would ensure that the OD620 values will fall within the standard curve range (10–100 µg/mL glucose).

### Timing

We routinely analyse 200 samples per week. The whole protocol can be broken into five parts with an overnight stopping point after each part.

Day 1, step 1—sample collection. Up to 6 h for 200 samples

Day 2, steps 2–6—AIR preparation with overnight drying. Up to 6 h for 200 samples

Day 3, steps 7–8—weighing of the sample and transfer to glass tubes. Up to 6 h for 200 samples

Day 4, steps 9–14—acetic nitric treatment with overnight drying. Up to 6 h for 200 samples

Day 5, steps 15–24—sulphuric acid swelling and anthrone assay. Up to 6 h for 200 samples.

## Troubleshooting

Troubleshooting advice can be found in Table 1.

**Table 1:** Troubleshooting guide

Steps	Problem	Possible reason	Solution
20	Anthrone solution not developing colour	Used 67 % sulphuric acid instead of conc. Sulphuric acid	Remake anthrone solution with conc. Sulphuric acid
20	Anthrone solution getting dark too quickly	Used sulphuric acid from an old bottle	Remake anthrone solution with conc. Sulphuric acid from a new bottle
23	OD620 values outside the standard curve range	Too much/too little sample used for anthrone assay	Use more/less amount of sulphuric acid treated sample

## Abbreviations

CSC: cellulose synthase complex
CESA: cellulose synthase
*irx*: *irregular xylem*
AIR: alcohol insoluble residue
PPE: personal protective equipment
